# Unravelling the Impact of Microgravity on Calcium Ion Signaling and Sensorium in Spaceflight

**DOI:** 10.3390/life16071096

**Published:** 2026-06-30

**Authors:** Lin Marza, Roula Mohammed, Yousif Abdelrahman, Abdullah Hajjiri, Malek Abuhjar, G. Roshan Deen

**Affiliations:** Materials for Medicine Research Group, School of Medicine, Royal College of Surgeons in Ireland (RCSI), Medical University of Bahrain, Busaiteen 228, Bahrain; 24200218@rcsi.com (L.M.); 24204690@rcsi.com (R.M.); 24200245@rcsi.com (Y.A.); 24200754@rcsi.com (A.H.); 24204663@rcsi.com (M.A.)

**Keywords:** microgravity, Calcium ion, signal transduction

## Abstract

Human spaceflight in microgravity induces profound physiological adaptations, yet its effects on the sensory system remain comparatively underexplored. While musculoskeletal and cardiovascular changes are well documented, sensory alterations pose equally important challenges to astronaut safety, performance, and post-mission recovery. Calcium ions (Ca^2+^), as universal intracellular messengers, play central roles in sensory transduction, neurotransmitter release, and adaptive signaling across all sensory modalities. Emerging evidence suggests that microgravity may influence Ca^2+^ homeostasis and Ca^2+^-dependent cellular processes, potentially affecting the functional integrity of sensory pathways. In this review, we synthesize current findings on the impact of microgravity on Ca^2+^-dependent processes in the five classical senses. Evidence from spaceflight studies, ground-based analogs, and related physiological models suggests possible alterations in taste receptor signaling, Ca^2+^-binding protein expression, mechanotransduction pathways, and vestibular function. However, direct evidence for microgravity-induced disruption of Ca^2+^ signaling remains limited for several sensory modalities. Collectively, these changes are associated with altered taste and smell perception, visual disturbances, reduced tactile sensitivity, and vestibular imbalance. By integrating both direct evidence and mechanistic hypotheses across sensory systems, this review highlights Ca^2+^ signaling as a potential unifying mechanism underlying sensory adaptation to microgravity. We further identify key knowledge gaps and discuss potential directions for developing targeted countermeasures aimed at preserving sensory function during long-duration missions. Beyond spaceflight, these insights contribute to a broader understanding of Ca^2+^-mediated sensory physiology under extreme environmental conditions.

## 1. Introduction

Human space exploration exposes astronauts to extreme environmental conditions, with microgravity being one of the most physiologically disruptive [1]. Microgravity is a state of near-weightlessness experienced during spaceflight as spacecraft and their occupants continuously free-fall around Earth. This environment leads to the loss of normal gravitational loading and profoundly alters cellular, physiological, and sensory processes. While the effects of microgravity on bone density, muscle mass, and cardiovascular function are well studied, its influence on the human sensory system remains a large gap in research. Sensory perception is key to astronaut performance. It aids in navigation, environmental awareness, nutrition, communication, and overall quality of life during missions. Any degradation in sensory function can risk safety and mission success [2]. Astronauts have reported sensory alterations during spaceflight, including visual disturbances, vestibular dysfunction and spatial disorientation, altered taste and smell perception, and impairments in sensorimotor performance, highlighting the vulnerability of sensory systems in microgravity.

The five primary senses—taste, smell, vision, touch, and hearing—share one unifying element, Ca^2+^. It is a universal secondary messenger critical for receptor activation, neurotransmitter release, and sensory adaptation [3,4]. Disruption of Ca^2+^ homeostasis is therefore a key consequence of exposure to microgravity. Microgravity disrupts Ca^2+^ homeostasis through several mechanisms. Microgravity may alter Ca^2+^ homeostasis through changes in cellular mechanotransduction and intracellular signaling. Under normal gravity, cells continuously experience mechanical forces that help regulate cytoskeletal organization, ion channel activity, and Ca^2+^-dependent signaling pathways. In microgravity, the loss of gravitational loading can modify the activity of mechanosensitive channels, disrupt cytoskeletal architecture, and alter intracellular Ca^2+^ transport and storage. These changes, together with microgravity-associated oxidative stress and fluid redistribution, may contribute to dysregulated Ca^2+^ signaling across multiple physiological systems [3,4]. While many existing studies focus on musculoskeletal and cardiovascular systems, emerging evidence indicates that sensory systems are equally vulnerable to Ca^2+^ signaling disturbances in microgravity [5,6].

The aim of this review is to integrate current evidence on how microgravity affects Ca^2+^-dependent mechanisms in human sensory systems. This knowledge is crucial for formulating effective strategies to protect astronaut sensory function during long-duration missions. While previous reviews focused on individual sensory systems under microgravity, to our knowledge, no comprehensive review has examined the role of Ca^2^ signaling across all five human senses. This narrative review synthesizes mechanistic findings related to vision, hearing, taste, smell, and touch, with particular emphasis on shared and system-specific Ca^2+^-dependent pathways. The review is organized by sensory modality and concludes by identifying common mechanistic themes, current knowledge gaps, and priorities for future research.

## 2. Methods

In this work, a focused narrative literature review was used to examine the effects of microgravity on Ca^2+^ ion signaling across human sensory systems, with a focus on taste, smell, vision, touch, and hearing. A narrative review was selected due to the diversity of experimental models, sensory systems, and outcome measures reported in the available literature. The objective was to integrate current evidence and experimental studies related to Ca^2+^ ion signaling mechanisms involved in human sensory perception in microgravity and simulated microgravity conditions.

### 2.1. Search Strategy and Data Extraction

A targeted literature search was conducted using PubMed, ScienceDirect, and Google Scholar. A combination of keywords such as “microgravity,” “spaceflight,” “Ca^2+^ signaling,” “calcium signaling,” “olfactory,” “gustatory,” “vision,” “touch perception,” “mechanotransduction,” “retina,” “vestibular,” “hearing,” and “sensory transduction,” was used. The search focused on studies with high relevance to Ca^2+^-based mechanisms involved in sensory function under microgravity or simulated microgravity environments. The search primarily focused on studies published between 2000 and 2025, although earlier foundational studies were considered where necessary to provide mechanistic context. Screening and study selection were conducted by the authors based on relevance to the review objectives and predefined inclusion criteria.

Studies were eligible for inclusion if they were peer-reviewed publications that investigated sensory systems or sensory-related cellular processes in the context of actual or simulated microgravity and reported findings related to calcium signaling pathways, calcium channels, calcium-binding proteins, intracellular calcium regulation, or downstream signaling mechanisms. Experimental studies involving human participants, cell cultures, animal models, and verified microgravity simulation systems were taken into consideration. Studies were excluded if they consisted only of conference abstracts, editorials, or non-peer-reviewed sources, did not address calcium-dependent pathways, or concentrated only on non-sensory physiological systems.

Initially, 132 records were identified and screened for relevance. After title, abstract, and full-text evaluation, 89 records were excluded. The final qualitative synthesis included 43 papers that satisfied the inclusion criteria. Foundational studies published before 2000 and selected review articles were additionally included where necessary in order to enhance the understanding of mechanistic findings and provide historical context. Pertinent information extracted from each study included the sensory modality investigated, experimental model, microgravity platform, calcium-related mechanisms examined, and principal findings.

Selected evidence was organized thematically by sensory modality and the molecular mechanisms related to Ca^2+^ regulation, including Ca^2+^ channels, Ca^2+^-binding proteins, intracellular signaling cascades, and transcriptional regulation. This organization allowed an integrative comparison of similarities and differences across sensory systems, highlighting convergent patterns of disrupted Ca^2+^ signaling during microgravity.

### 2.2. Analysis

Given the heterogeneity of experimental models, study designs, microgravity platforms, and outcome measures reported across the literature, quantitative synthesis was not considered appropriate. Instead, a qualitative comparative analysis was performed to identify common mechanistic trends, areas of agreement and knowledge gaps related to calcium-dependent sensory signaling under microgravity conditions.

Although narrative reviews are inherently limited by the lack of formal quantitative analysis and potential selection bias, this narrative synthesis provides a comprehensive way to examine the potential effects of gravitational environments on calcium-mediated sensory signaling pathways.

## 3. Results

### 3.1. General Cellular and Molecular Mechanisms of Cytosolic Ca^2+^ Signaling

At the cellular and molecular level, cytosolic Ca^2+^ changes are a central mechanism by which cells convert stimulation into a biological response. Under resting conditions, cytosolic Ca^2+^ is maintained at a very low concentration of around 100 nM, while extracellular Ca^2+^ is approximately 1.2 mM, creating a steep concentration gradient across the plasma membrane. When a cell is stimulated, Ca^2+^ can enter the cytosol through plasma membrane Ca^2+^ channels or be released from intracellular stores such as the endoplasmic reticulum through IP3 receptors and ryanodine receptors. This increase in cytosolic Ca^2+^ is detected by Ca^2+^-binding proteins, including EF-hand proteins such as calmodulin, which undergo conformational changes and activate downstream signaling pathways. The effect of a Ca^2+^ signal depends on its size, duration and location within the cell. After signaling, cytosolic Ca^2+^ is returned toward resting levels through buffering and clearance mechanisms such as mitochondria, Ca^2+^ ATPase pumps, sodium–calcium exchangers and reuptake into intracellular stores. Therefore, disruption of Ca^2+^ entry, release, buffering, or clearance can affect the strength of cellular signaling responses [3].

### 3.2. Role of Ca^2+^ in Taste, the Gustatory System, and Ca^2+^-Sensing Receptor (CaSR)

The gustatory system is the system responsible for detecting and interpreting taste and flavor, such as sweet, salty, sour, bitter and umami [7]. Ca^2+^ channels and receptors regulate and sense the levels of Ca^2+^ in the body. According to the “Involvement of the Ca^2+^-sensing Receptor in Human Taste Perception”, it has been found that Ca channels are closely related to receptors that sense sweet and sour tastes. Despite being an older study, it is included as it provides foundational human evidence that CaSR activation modulates taste perception, and the core receptor mechanism remains consistent in later structural/mechanistic research. This study investigated whether the CaSR which is a G-protein coupled receptor (GPCR) known for maintaining Ca homeostasis is functionally involved in human taste perception especially in enhancing different forms of taste such as sweet, salty, sour and umami [8]. The structure consists of a membrane-bound Class C GPCR whose function relies on ligand-induced conformational shifts [9]. Although direct spaceflight evidence linking microgravity to calcium-dependent taste transduction remains limited, it is reasonable to hypothesize that microgravity-mediated disruption of calcium homeostasis and CaSR-mediated signaling in taste receptor cells could affect or change the intracellular Ca^2+^ dynamics, neurotransmitter release, and ultimately distort taste perception. This is a fair hypothesis because the activation of the receptor correlates with the measurable changes in perception of taste [8].

#### 3.2.1. Role of Ca^2+^ in Taste Signal Transduction

As previously mentioned, the gustatory system relies on Ca^2+^ to play a role in signal transduction across the different forms of taste (taste modalities) [7]. Although Medler’s review does not directly investigate microgravity, it provides a foundational explanation of Ca^2+^-dependent taste transduction, including Ca^2+^ influx, Ca^2+^ release from internal stores, mitochondrial buffering, and sodium/calcium exchanger regulation. Based on this framework, it is reasonable to hypothesize that microgravity-related disruption of calcium homeostasis could impair the formation or regulation of taste-evoked Ca^2+^ signals, thereby altering neurotransmitter release and taste perception [10]. Normally, salt taste is mediated by epithelial sodium channels that allow sodium to enter the taste cell, leading to depolarization and the opening of voltage-gated Ca^2+^ channels, which trigger serotonin release and activate sensory neurons [7]. Medler also explains that salt taste helps detect ions essential for maintaining the body’s electrolyte balance [10]. Sour taste perception similarly involves depolarization due to hydrogen ions, which open voltage-gated Ca channels, resulting in serotonin release [7]. Medler further suggests that sour taste also involves sensing various ions, along with salty taste [10], which are referred to as ionic stimuli. In contrast, bitter, sweet, and umami tastes are all mediated by GPCRs that use a second messenger system to increase intracellular Ca^2+^, either by releasing it from internal stores or enhancing Ca^2+^ entry, which suggests why Medler refers to them as “chemically complex” [10]. This rise in Ca^2+^ levels leads to ATP release, which stimulates afferent neurons [7].

#### 3.2.2. Effect of Microgravity on Ca^2+^ and Taste

Given that Ca^2+^ influx or mobilization is a very important step in all five taste forms, any disruption in Ca^2+^ signaling could affect taste perception [7]. Therefore, a hypothesis based on this information can be made suggesting that alterations in Ca^2+^ homeostasis or the function of Ca-sensitive receptors, such as the CaSR, caused by microgravity, may impair intracellular Ca^2+^ signaling in taste receptor cells. As a result, the release of neurotransmitters like serotonin or ATP could be diminished, leading to reduced or distorted perception of saltiness, sourness, bitterness, sweetness, and umami flavors during spaceflight.

As mentioned in Section 3.1, the structure of CaSR is a membrane-bound Class C GPCR whose function relies on ligand-induced conformational shifts, Although direct evidence on CaSR behavior in taste receptor cells under microgravity is limited, a study on cultured myocytes after spaceflight showed that microgravity can change the cytoskeletal organization and reduce receptor aggregation [11]. While this evidence is not direct because it was gathered from myocytes rather than taste cells, it remains relevant because it demonstrates that spaceflight can disrupt cellular structures that help organize membrane-associated receptors. Therefore, it is reasonable to hypothesize that microgravity-related changes in membrane and cytoskeletal organization could affect the stability, localization or even the signaling efficiency of receptors such as CaSR, potentially impairing downstream taste transduction and contributing to altered taste perception during or after spaceflight.

#### 3.2.3. Disruption of Ca^2+^ Metabolism in Oral Tissues Under Microgravity

A study on rhesus monkeys exposed to two weeks of spaceflight revealed significant increases in intracellular Ca^2+^, phosphorus, and potassium in sublingual epithelial cells compared to control conditions [12]. Although this study is older and does not directly investigate taste receptor cells or taste perception under microgravity, it provides rare direct spaceflight evidence that microgravity can alter ionic balance in oral tissues. This is relevant as Ca^2+^ signaling is essential for neurotransmitter release across all five basic taste modalities, meaning that any disruption of Ca^2+^ metabolism in oral tissues could indirectly affect signaling in the gustatory system. Therefore, it supports the broader hypothesis that microgravity-related changes in oral Ca^2+^ and ionic balance may contribute to altered taste perception by affecting receptor activation, intracellular Ca^2+^ signaling, or even synaptic transmission.

#### 3.2.4. Environmental Influences on Flavor Perception in Simulated Microgravity

In addition to molecular mechanisms, environmental conditions unique to spaceflight also contribute to altered taste perception. A 2024 study by RMIT University investigated how the perception of food aromas changes in simulated microgravity using virtual reality environments designed to mimic the International Space Station (ISS). Researchers found that participants reported significantly stronger perceptions of vanilla and almond aromas (which fall under sweet modalities) under space conditions, while lemon (a sour modality) remained unaffected [13]. These findings suggest that sensory modulation in microgravity is not only biochemical but also psychological and environmental, potentially influenced by being enclosed in a limited physical space, reduced airflow, and emotional state. Although the study focused on aroma rather than taste receptor activation, it illustrates the broader impact of spaceflight on food perception.

These environmental factors may interact with or exacerbate molecular disruptions such as those involving Ca^2+^ signaling, further impairing the normal function of taste receptors like CaSR during space missions. While the RMIT study [13] provides valuable insight into how space environments affect aroma perception, it does not directly investigate the molecular mechanisms of taste, such as Ca^2+^ signaling or receptor activation. The study focuses primarily on olfactory responses rather than gustatory pathways, and the simulated microgravity conditions achieved through virtual reality do not fully replicate the physiological effects of actual spaceflight. Therefore, while the findings are useful for understanding environmental influences on flavor perception, they should be interpreted as complementary rather than conclusive evidence for biochemical changes such as Ca^2+^’s role in taste transduction under microgravity.

#### 3.2.5. Simulated Microgravity Increases Taste Intensity Despite Reduced Aroma Perception

Building on the molecular hypothesis that microgravity may disrupt Ca^2+^-dependent signal transduction (including CaSR-linked pathways) in taste receptor cells, behavioral evidence from a controlled stimulated microgravity study suggests that perceived taste intensity can increase even when olfactory input decreases. In Gonzalez Viejo et al. (2024) [14], trained panelists (n = 12 for basic tastes and n = 14 for aromas) evaluated ISO-prepared taste solutions: sweet (sucrose 12 g/L), salty (NaCl 2 g/L), sour (citric acid 0.60 g/L), bitter (caffeine 0.27 g/L), and umami (MSG 1 g/L) across three body positions which are: normal sitting (90° to 270°), semi-reclined (55° to 135°), and a “microgravity” posture (0° to 170°, in which the legs were elevated above heart level). Using a 15 cm intensity scale, overall taste intensity was much higher in the microgravity position, with a mean of 7.19, than in the normal position, with a mean of 5.27, while the aroma intensity showed the opposite pattern, with a mean of 7.24 in the microgravity position was and 8.96 in the normal position. These findings are consistent with microgravity fluid shifts that reduce odorant delivery to nasal receptors.

Physiological biometrics concurrently shifted with posture, resulting in decrease in heart rate and blood pressure with reclining, for example during the aroma session, the heart rate decreased from 96 BPM in normal sitting to 87 BPM in microgravity and the systolic pressure from 119 to 103 mmHg, the diastolic pressure fell from 78 to 73 mmHg. Together these results support the idea that spaceflight-associated taste changes are not purely perceptual or environmental, but microgravity-related physiological shifts can coincide with increased gustatory intensity despite the reduced olfaction this aligns with the Ca^2+^ focused framework by motivating targeted mechanistic testing whether altered ionic homeostasis and downstream intracellular Ca^2+^ handling including CaSR-mediated modulation, contribute to the amplified taste signaling reported under microgravity conditions or conditions similar to it [14].

The evidence presented across this research highlights the essential role of Ca^2+^ signaling in taste perception and how this process may be disrupted by microgravity. Ca^2+^ acts as a critical messenger across all five basic taste modalities, and receptors such as the CaSR play a key role in sensing and modulating taste responses. Under microgravity, structural changes to cell membranes, altered Ca^2+^ homeostasis, and cytoskeletal disorganization could impair receptor function, thereby diminishing taste signal transduction. Additionally, environmental and psychological factors in space, such as confinement and reduced airflow, may further exacerbate these disruptions. While some findings, like those from simulated microgravity studies, are indirect or based on aroma rather than taste, they nonetheless highlight the complex and multifactorial nature of taste alterations in space. Together, these insights support the hypothesis that microgravity can negatively impact taste perception through both molecular and environmental mechanisms, requiring further investigation into this area of study, especially in long-duration space missions where nutrition and sensory satisfaction are vital for astronaut physical health, mental health and well-being.

Although direct spaceflight data on Ca^2+^’s role in gustation are scarce, parallels with olfactory and neuronal Ca signaling suggest similar vulnerabilities, underscoring this as a key research gap for future study. Like taste, smell also relies on precise Ca^2+^ signaling and here too, microgravity seems to weaken both amplification and adaptation.

### 3.3. Role of Ca^2+^ in Olfactory Signal Transduction

#### 3.3.1. Ca^2+^ Entry and Dual Function in Olfactory Cilia

Ca^2+^ ions are central to olfactory signal transduction, operating within the highly compartmentalized environment of olfactory cilia where cyclic nucleotide-gated channels, Ca^2+^-activated chloride channels, and Ca^2+^-binding proteins interact to shape odor detection and adaptation [15]. When an odorant binds to receptors on the cilia of an ORN, it sets off a cAMP-based signaling cascade that opens cyclic nucleotide-gated (CNG) channels [16]. This allows Ca^2+^ to rush into the cell. That initial burst of Ca^2+^ into the ciliary cytoplasm has two purposes: it boosts the electrical signal by activating Ca^2+^-dependent chloride (Cl^−^) channels, and it activates negative feedback mechanisms that help the neuron adapt to continuous stimulation so that it does not keep firing in response to the same odor [16,17].

#### 3.3.2. Segregated Timing of Amplification and Adaptation

**Figure 1:**
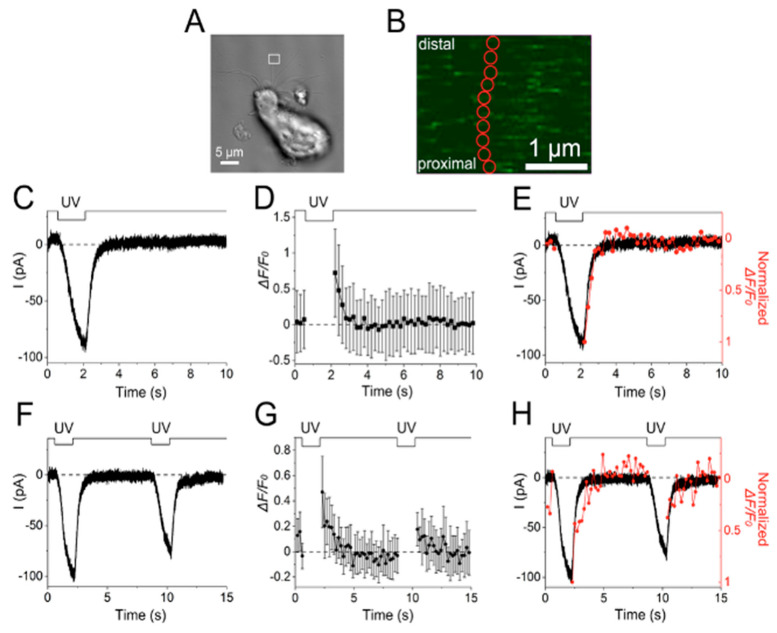
Time-course graphs showing Ca^2+^ signals and membrane currents in olfactory cilia. (**A**) Differential interference contrast image of an isolated olfactory cilium. (**B**) Fluorescence image showing Ca^2+^ imaging along the cilium. (**C**) Representative membrane current recording following UV-induced stimulation. (**D**) Corresponding Ca^2+^ response (ΔF/F_0_). (**E**) Simultaneous recording of membrane current (black) and normalized Ca^2+^ signal (red). (**F**–**H**) Representative responses during repeated stimulation illustrating temporal dissociation between Ca^2+^ signaling and electrical adaptation. Black traces indicate membrane currents, red traces indicate normalized Ca^2+^ signals, and green fluorescence images represent Ca^2+^ imaging within olfactory cilia. Reproduced from Ref. [16].

A study by Takeuchi and Kurahashi (2022) looked at these mechanisms in depth. Using isolated olfactory cilia from newts, they combined high-resolution Ca^2+^ imaging with patch-clamp recordings [16]. By triggering cAMP release with a UV laser and tracking Ca^2+^ with Fluo-4 dye, they observed a sharp spike in Ca^2+^ just after stimulation, which corresponded with a quick burst of Cl^−^ current [16]. Even after Ca^2+^ levels settled back to baseline, the adaptive response continued [16]. This suggests that while signal boosting through Cl^−^ channels is linked to brief Ca^2+^ spikes, adaptation may be sustained through longer-lasting interactions between Ca^2+^ and proteins such as calmodulin [16]. Takeuchi and Kurahashi’s work (Figure 1) demonstrated that these two processes—amplification and adaptation—occur in the same small space but appear to operate on different time scales [16]. It is also worth noting that they avoided using artificial Ca^2+^ buffers (like EGTA) in some trials, which made their data more physiologically relevant [16].

A time-course graph showing the Ca^2+^ signal (green) and the membrane current (black) indicates that the Ca^2+^ concentration returns to baseline immediately after stimulation, while the adaptive electrical response continues. This illustrates how signal amplification and feedback inhibition in olfactory cilia may be temporally dissociated.

#### 3.3.3. Functional Role of Ca^2+^-Dependent Cl^−^ Currents

Complementing these findings, research by Reisert and Reingruber (2016) provided evidence that Ca^2+^-activated Cl^−^ channels contribute importantly to maintaining reliable and strong olfactory signals [17]. Using mouse olfactory neurons, they highlighted that when these Cl^−^ channels were blocked, odor-evoked responses became weaker and less consistent, specifically when stimuli were repeated in rapid succession [17]. These findings suggest that Ca^2+^-dependent Cl^−^ currents contribute substantially to the generation of quick and precise signaling in ORNs [17].

**Figure 2:**
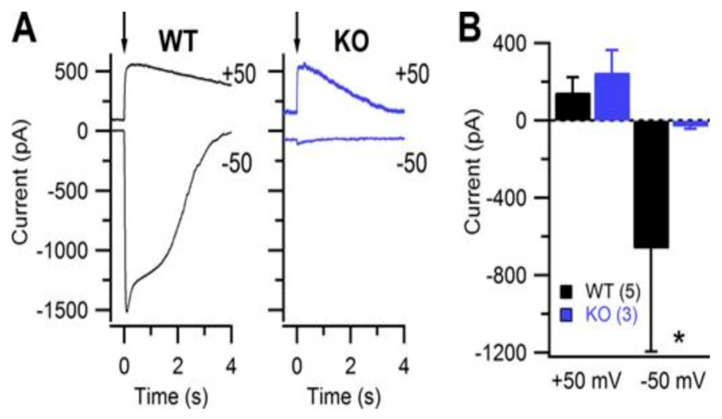
Comparison of odor-evoked responses in olfactory receptor neurons under normal conditions (WT) and when activated Ca -activated Cl^−^ channels are inhibited or absent (KO). (**A**) Representative current traces recorded from wild-type (WT) and knockout (KO) olfactory receptor neurons following odor stimulation at depolarized (+50mV) and hyper polarized (−50mV) membrane potentials. Arrows indicate the time of odor stimulation. (**B**) Quantification of odor-evoked current amplitudes in WT and KO neurons, highlighting reduced responses in knockout cells. The asterisk (*) denotes a statistically significant difference between wild-type (WT) and knockout (KO) responses (*p* < 0.05). Image reprinted from Ref. [17].

This figure (Figure 2) compares odor-evoked responses in olfactory receptor neurons under normal conditions (WT) and when Ca^2+^-activated Cl^−^ channels are inhibited or absent (KO).

In Panel A, the current traces highlight that wild-type neurons generate strong inward currents following stimulation, while knockout (KO) neurons exhibit noticeably reduced responses. These findings suggest that the absence of Ca^2+^-activated Cl^−^ channels is associated with reduced signal amplitude.

In Panel B, the findings are quantified and show a substantial reduction in current magnitude at both depolarized (+50 mV) and hyperpolarized (−50 mV) potentials in knockout cells compared to wild-type.

These findings indicate that Ca^2+^-activated Cl^−^ channels play an important role in amplifying odor-evoked signals. Without this Ca^2+^-dependent amplification mechanism, odor-evoked responses may become weaker and less reliable, particularly during repeated stimulation.

#### 3.3.4. Calmodulin as the Molecular Switch

Kaneko and colleagues (2006) examined specific mechanisms by which Ca^2+^ stimulates Cl^−^ channels, which provided additional molecular understanding [18]. Their work suggested that Ca^2+^ binds to calmodulin rather than opening these channels directly. Instead, it interacts with the channel to permit gating [18]. By mutating specific calmodulin sites, the researchers displayed that interrupting this interaction reduced the channel’s response to Ca^2+^ [18]. This finding supports an important role for Ca^2+^–protein interactions in shaping olfactory signal strength [18]. While the study dates to 2006, more recent research, such as that of Takeuchi and Kurahashi (2022), which directly observed structural and functional segregation of Ca^2+^ effects within olfactory cilia, has strengthened support for these conclusions [16]. Together, these studies support an important role for Ca^2+^-CaM interactions in signal amplification [16,18].

When calmodulin’s ability to bind Ca^2+^ is compromised, sensitivity curves comparing wild-type and mutant calmodulin demonstrate a marked decrease in Cl^−^ current. These findings suggest that Ca^2+^ signaling in olfactory neurons serves two distinct functions—one immediate and localized, improving the signal through chloride channel activation, and the other longer-lasting, regulating adaptation through protein interactions such as with calmodulin [16,17,18]. This division of functionality illustrates the highly regulated nature of Ca^2+^ signaling within olfactory cilia, both in time and space [16,18]. Because this system is tightly controlled, even slight microgravity-induced shifts could interfere with how odors are detected and processed in space [16,19,20,21].

#### 3.3.5. Influence of Gravity on Neuronal Firing and Ca^2+^ Dynamics

**Figure 3:**
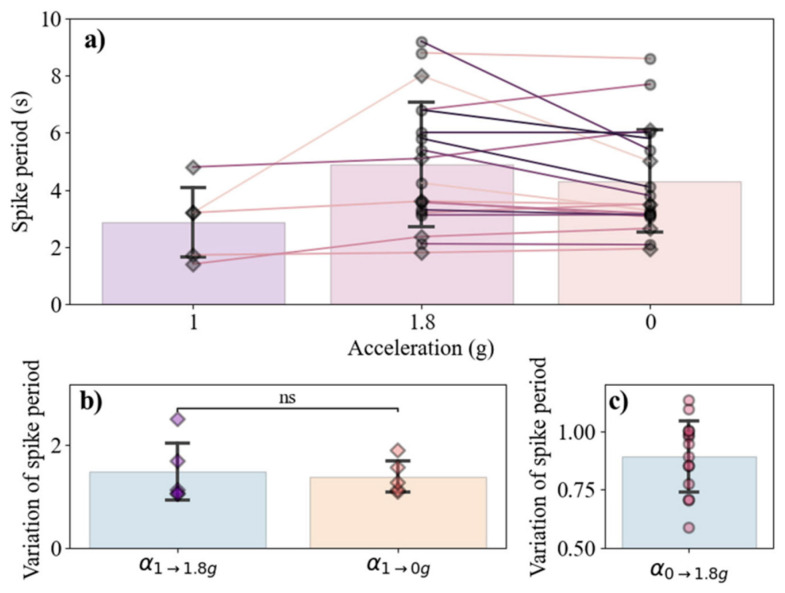
Calcium activity in primary hippocampal neurons during parabolic flight. (**a**) Spike periods of active neurons measured during 1 g, 1.8 g, and µg phases. Diamonds represent neuronal groups active throughout the parabola, whereas circles represent groups that began firing during. the 1.8 g phase. Linked points correspond to the same neuronal group. (**b**) Relative spike period variation compared with the 1 g phase for neurons already active at 1 g. (**c**) Relative spike period vaiation between the 1.8 g and µg phases in neurons inactive at 1 g. "ns" indicates a non-significant difference. Image reprinted from Ref. [19].

Microgravity environments have been associated with alterations in intracellular Ca^2+^ regulation in neurons, potentially influencing their excitability, gene expression patterns, and synaptic activity [19]. A recent study by Lecoq et al. (2024) used Ca^2+^ imaging of primary hippocampal neurons during parabolic flight to examine how brief gravitational changes affect neuronal signaling [19]. Neurons that remained inactive under Earth gravity (1 g) began to fire spontaneously under microgravity (µg), while others showed prolonged interspike intervals, reflecting a slowing of Ca^2+^ oscillation patterns Figure 3 [19]. The findings suggest that gravity may influence baseline neuronal activity by modulating intracellular Ca^2+^ dynamics [19].

These panels show signs of Ca^2+^ activity in both normal (1 g) and microgravity (µg) settings. Changes in neuronal Ca^2+^ activity were observed under µg conditions, where previously silent neurons began firing and spike intervals grew. Transcriptomic evidence further suggests that microgravity may alter Ca^2+^-dependent regulatory networks at the genomic level [20]. Abdelfattah et al. (2024) conducted a broad omics analysis across multiple cell types exposed to simulated microgravity and identified significant downregulation of Ca^2+^-responsive transcriptional pathways [20]. One example is the reduced activity of the nuclear factor of activated T cells (NFAT), a transcription factor controlled by the Ca^2+^ influx through the calcineurin pathway [20]. The researchers found that while NFAT itself remained structurally undamaged, its DNA-binding activity was impaired due to downregulation of its transcriptional co-activator, AP-1, under µg conditions [20]. This disconnection indicates that even when Ca^2+^ is successfully mobilized within the neuron, its downstream influence on gene regulation may be restrained [20].

#### 3.3.6. Functional and Genomic Disruption in Neural Cells

These findings suggest that neurons exposed to microgravity may experience changes in both cellular function and gene regulation [19,20]. Microgravity may disrupt neurons on two fronts: by altering their firing patterns and Ca^2+^ activity, and by influencing gene expression pathways that respond to Ca^2+^ signals [19,20]. While these mechanisms have been documented in hippocampal and progenitor neurons, it is reasonable to hypothesize that similar vulnerabilities may occur in other sensory neurons, such as olfactory receptor neurons. This is because olfactory signal neurons similarly rely on tightly regulated Ca^2+^ signaling for rapid detection and adaptive feedback mechanisms [16,19,20,21]. Importantly, these observations were obtained from hippocampal neurons and other neural cell populations rather than olfactory neurons directly.

#### 3.3.7. Ca^2+^ Signaling Proteins Are Downregulated in Microgravity

While research on neural progenitors and hippocampal neurons provides evidence that Ca^2+^-dependent signaling pathways may be vulnerable to microgravity, their relevance to olfactory function remains indirect [21]. Han et al. investigated gene expression in mouse neural stem cells subjected to both real spaceflight and simulated microgravity. Their transcriptomic analysis showed significant downregulation of several Ca^2+^-dependent signaling components, including calmodulin (CaM) and Ca^2+^/calmodulin-dependent protein kinase II (CAMKII) [21]. These proteins play important roles in olfactory receptor neuron (ORN) adaptation, where Ca^2+^-CaM binding contributes to the feedback inhibition involved in sensory desensitization to persistent odorants [16,21].

#### 3.3.8. A Broader Pattern of Suppression Across Cell Types

This pattern of molecular changes raises the possibility that shared mechanisms may contribute to Ca^2+^ signaling suppression in microgravity [20,21]. It aligns with the gene suppression patterns observed by Abdelfattah et al., who found similar downregulation across various stem and progenitor cell lines, including mesenchymal and neural cells [20]. Since ORNs rely on coordinated interactions between CNG channels, calmodulin, and CAMKII for signal amplification and adaptation, reduced expression of these proteins may lead to decreased feedback inhibition, increased noise in the sensory signal, or total desensitization [16,20,21]. However, these effects have not been directly demonstrated in olfactory neurons under microgravity conditions.

#### 3.3.9. Implications for Olfactory Processing in Space

Although olfactory neurons were not directly examined in these studies, it is plausible that they may share some of the molecular vulnerabilities observed in other neuronal populations exposed to microgravity [20,21]. Since olfactory cilia depend on highly localized Ca^2+^ signals, even simple disruptions to proteins like calmodulin or CAMKII could interfere with normal sensory processing [16,21]. Therefore, these findings raise the possibility that microgravity-induced modifications in Ca^2+^ signaling may also affect olfactory sensory integrity [16,20,21].

Although direct studies on olfactory receptor neurons (ORNs) in space are lacking, converging evidence from behavioral, molecular and anatomical studies suggests that microgravity conditions can compromise olfactory function [22]. One of the earliest insights into this plausibility comes from Olabi et al. (2002), who reviewed NASA post-mission reports and found that “astronauts have reported diminished, unchanged, and occasionally heightened smell and taste sensations during spaceflight” [22]. Although the data are largely anecdotal, they are consistent with the possibility of physiological changes in the chemical senses during spaceflight and suggest that microgravity may influence olfactory perception [22].

#### 3.3.10. Structural Changes in the Olfactory Bulb

Structural evidence provides additional support for this possibility. In a spaceflight study by Stodieck et al. (2015), mice were flown aboard the STS-135 mission and showed reduced neurogenesis in the olfactory bulb, along with slight reductions in overall bulb volume, relative to ground-based controls [23]. The olfactory bulb is responsible for the first level of odor signal processing in the brain, therefore, alterations in its structure or neurogenic capacity could potentially translate to sensory deficits [23]. Neurogenesis in this region involves complex developmental processes such as synaptogenesis and axonal pruning. Therefore, these structural changes may be consistent with the Ca^2+^ imbalance observed under microgravity, although a direct causal relationship has not been established [19,20,21,23]. Moreover, decreased expression of neurogenic markers such as NeuN and BrdU+ was reported, providing a histological basis for impaired olfactory plasticity and neurogenesis [23].

#### 3.3.11. Gene-Level Disruptions Suggest Altered Ca^2+^ Signaling Pathways

At the cellular level, both Abdelfattah et al. (2024) and Han et al. (2021) provide transcriptomic data suggesting that several Ca^2+^-regulated signaling pathways are downregulated under microgravity conditions in a range of neural and stem cell types [20,21]. Specifically, studies by Abdelfattah et al. (2024) found suppression of NFAT (nuclear factor of activated T-cells), a transcription factor activated by Ca^2+^ that plays an important role in cellular adaptation and gene expression [20]. Similarly, Han et al. highlighted the suppression of important signaling components such as calmodulin and CAMKII—proteins that are also necessary for olfactory adaptation and feedback regulation [21]. Given our understanding of Ca^2+^ signaling in the olfactory system, these molecular changes raise the possibility that multiple stages of olfactory processing—detection, amplification, and adaptation—could be influenced in space. Such disruption could explain why smell perception becomes less reliable in space [20,21,22].

#### 3.3.12. Cumulative Evidence of Olfactory Impairment in Space

In conclusion, available evidence suggests that microgravity may influence both the structural integrity of the olfactory bulb and the molecular machinery responsible for Ca^2+^-mediated signal processing [20,21,22,23]. Together, the altered neuronal behavior, changes in Ca^2+^-regulated gene pathways, and astronaut reports are consistent with the possibility that olfactory function may be impaired under microgravity [20,21,22,23]. Such impairments could affect both the well-being of astronauts and their ability to carry out important tasks like detecting environmental hazards [22,23]. Similar alterations in Ca^2+^- regulatory pathways have been reported in other sensory systems, including vision, where changes in Ca^2+^-binding proteins may contribute to increased cellular vulnerability.

### 3.4. Role of Ca^2+^ in Vision

#### 3.4.1. Phototransduction and Ca^2+^ Regulation in the Retina

Phototransduction is the process by which light is converted into an electrical signal in the retina, allowing the brain to generate a visual image. This process takes place in photoreceptors known as rods and cones, which are located in the outermost layer of the retina. A key regulator of phototransduction is Ca^2+^, which acts as a secondary messenger to help control the sensitivity of photoreceptors to varying light intensities through a process known as light adaptation [24]. Under Earth’s gravity, Ca^2+^ homeostasis in the outer segments of photoreceptors is tightly maintained by a dynamic equilibrium involving a carefully regulated balance between Ca^2+^ influx and efflux mechanisms [24]. This Ca^2+^ balance is essential for visual signaling and ensures the survival of photoreceptors in the long term [24].

Rods and cones differ in their phototransduction cascades, and these differences are influenced by differences in Ca^2+^ homeostasis within each photoreceptor type. In darkness, cyclic nucleotide-gated (CNG) channels in the outer segments of photoreceptors remain open, allowing a continuous influx of sodium (Na^+^) and Ca^2+^ ions. This inward flow is known as the “dark current.” It maintains the photoreceptor in a depolarized state and enables continuous glutamate release at the synaptic terminal. Upon light exposure, activation of the phototransduction cascade leads to a decrease in cyclic GMP (cGMP), the molecule responsible for keeping CNG channels open. As a result, these channels close, reducing Ca^2+^ influx. However, Ca^2+^ extrusion from the outer segment continues, leading to a rapid decline in intracellular Ca^2+^ concentration and producing a hyperpolarized state [24].

#### 3.4.2. Ca^2+^-Dependent Feedback Mechanisms

Ca^2+^’s effects on phototransduction are mainly controlled by Ca^2+^-binding proteins such as GCAPs and recoverin, which help regulate photoreceptor sensitivity, intracellular Ca^2+^ levels, and response speed [24]. GCAP1 and GCAP2 act as Ca^2+^ sensors. In darkness, when Ca^2+^ levels are high, they inhibit the enzymes RetGC1 and RetGC2, leading to decreased cGMP synthesis and closure of CNG channels. In light, when Ca^2+^ levels fall, GCAPs activate these enzymes, increasing cGMP production and allowing CNG channels to reopen. Another Ca^2+^-binding protein, recoverin, controls rhodopsin kinase, the enzyme that deactivates the light-activated pigment rhodopsin. Under high Ca^2+^ conditions (dark), recoverin inhibits rhodopsin kinase, delaying signal shutoff. Under low Ca^2+^ conditions (light), this inhibition is released, enabling rhodopsin deactivation [24]. Together, these feedback mechanisms help turn off the light response and allow photoreceptors to adapt across a wide range of light conditions [24]. While both rods and cones rely on GCAPs and recoverin for Ca^2+^-dependent feedback, the response in cones is faster and more dynamic. This is due to cones having smaller outer segment volumes, faster Ca^2+^ extrusion rates, and additional Ca^2+^ regulation pathways [24]. As a result, cones are more efficient at rapid light adaptation, especially under bright and changing light conditions [24].

#### 3.4.3. Ca^2+^ Extrusion via Nckx Transporters

Restoring Ca^2+^ levels after light exposure is essential for maintaining Ca^2+^ homeostasis in photoreceptors. This task is carried out by specialized Ca^2+^ extrusion proteins known as NCKX transporters [24]. In rods, this is handled by the NCKX1 isoform, whereas cones rely on both NCKX2 and the cone-specific isoform NCKX4. These exchangers use sodium (Na^+^) and potassium (K^+^) gradients to actively pump Ca^2+^ out of the outer segment, enabling fast Ca^2+^ removal and promoting rapid light adaptation [24].

#### 3.4.4. Functional Role of Nckx4 in Cone Photoreceptors

Recent studies have shown the importance of NCKX4 in cone photoreceptors [25]. While it was previously thought that cones rely solely on NCKX2 for Ca^2+^ extrusion, research has now shown that NCKX4 plays a more abundant role in the rapid clearance of Ca^2+^ after light exposure [25]. Both transporters help restore Ca^2+^ homeostasis, but NCKX4 is more cone-specific and acts faster [25]. Electrophysiological studies in NCKX4-deficient mice showed delayed response recovery, impaired adaptation to high-frequency flickering light, and reduced visual performance under daylight conditions, showing its essential role in cone-mediated light adaptation [25]. Thus, under normal gravity conditions, Ca^2+^ signaling in the retina is tightly controlled by specialized transporters and feedback proteins, enabling rapid and sensitive visual function [24,25].

#### 3.4.5. Microgravity-Induced Retinal Damage and Apoptosis

Structural damage in the retina, particularly in vascular endothelial cells, has been shown to occur because of microgravity during spaceflight [26]. Microgravity exposure induces increased oxidative stress and elevated levels of lipid peroxidation, both of which are known to contribute to retinal cell damage [26]. In a mouse model, researchers observed a significant increase in apoptosis within the retinal tissue of spaceflight-exposed mice compared to ground controls [26]. This was confirmed using TUNEL staining, which showed a 64% rise in the number of apoptotic cells in the microgravity group [26]. Critically, when mice were exposed to artificial gravity during flight, the number of apoptotic cells was significantly reduced. This indicates that microgravity—not spaceflight alone—was the primary factor driving retinal damage and, potentially, visual impairment or neurodegeneration [26].

#### 3.4.6. Disruption of Retinal Proteomic and Molecular Pathways

In addition to structural damage, microgravity also disrupts key molecular pathways in the retina [26]. Proteomic analysis revealed that more than 250 proteins were differentially expressed in spaceflight-exposed mice [26]. These included proteins involved in cell survival, oxidative stress, mitochondrial function, and tissue repair [26]. While 3174 proteins were commonly expressed across all groups, the differences were primarily in expression levels rather than in their presence or absence [26].

Notably, the study identified downregulation of CLIP2, a cytoskeletal protein found in the nervous system that is important for maintaining retinal structure and integrity [26]. In contrast, MECP2, a neurodegenerative gene regulator associated with cell death and neurodegeneration, was found to be upregulated [26]. These findings demonstrate that microgravity causes structural and molecular alterations in the retina. Because Ca^2+^ signaling is essential for retinal homeostasis and phototransduction, these changes may influence Ca^2+^-dependent pathways; however, direct evidence of retinal Ca^2+^ dysregulation under microgravity is currently lacking.

#### 3.4.7. Ca^2+^-Binding Protein Dysregulation in Microgravity and Its Impact on Visual Signaling

While Section 1 highlighted how Ca^2+^ acts as a key messenger in phototransduction, this section focuses on the proteins that help maintain Ca^2+^ balance in retinal neurons: the Ca^2+^-binding proteins (CBPs). These proteins, such as Calbindin (CaB), Calretinin (CaR), and Parvalbumin (PV), play crucial roles in maintaining Ca^2+^ homeostasis in retinal neurons. They buffer intracellular Ca^2+^, influence neuronal excitability, and facilitate signal transmission across retinal circuits [27]. Each protein is unique in its function. CaB is mainly found in horizontal and some bipolar cells; it helps manage increases in Ca^2+^ concentration during neurotransmission. CaR is present in retinal cells and has multiple Ca^2+^-binding sites, making it particularly effective at buffering rapid changes in Ca^2+^. PV is mostly found in ganglion and displaced amacrine cells; it helps stabilize Ca^2+^ levels during fast, repeated signaling [27]. Together, these proteins work in coordination to keep Ca^2+^ levels within a healthy range, protecting neurons from stress and supporting accurate visual signal processing [27].

#### 3.4.8. Effects of Microgravity on Cbp Expression

Studies suggest that microgravity and spaceflight-related stress can lead to changes in the expression of CBPs in the retina. Under similar stress conditions such as ischemia, diabetic retinopathy, and glaucoma, the expression levels of CaB, CaR, and PV have been shown to decrease in retinal neurons, particularly within the inner nuclear layer (INL) and ganglion cell layer (GCL) [27]. These regions include important cells, such as bipolar, amacrine, and ganglion cells, which are essential for signal transmission. This reduction in CBP expression weakens the cell’s ability to buffer Ca^2+^ effectively, resulting in Ca^2+^ overload, which can lead to oxidative damage and potential cell death [27]. While direct studies on CBPs in space conditions are limited, related models demonstrate clear signs of Ca^2+^ dysregulation, oxidative stress, and structural changes in the retina during and after spaceflight exposure [28]. These findings support the possibility that CBPs are altered by microgravity, and their disruption may lead to weakened Ca^2+^ homeostasis, which in turn increases retinal vulnerability. Nevertheless, whether microgravity directly alters CBP expression and retinal Ca^2+^ homeostasis remains a hypothesis requiring experimental validation.

#### 3.4.9. Implications for Ca^2+^ Signaling and Visual Function

When proteins like CaB, CaR, and PV are downregulated, the retina becomes less capable of controlling Ca^2+^ fluctuations, resulting in dysregulated signaling and increased cellular stress [27]. Spaceflight is already known to cause oxidative stress and retinal structural damage, both of which may influence Ca^2+^-dependent cellular processes in the retina [28]. The additional loss of Ca^2+^-buffering capacity can exacerbate these consequences. This might lead to abnormal neurotransmission, impaired synaptic function, and ultimately, damage to the retinal neurons responsible for vision [27,28]. This mechanism represents one of the most direct links between microgravity and Ca^2+^ signaling imbalance and may contribute to visual symptoms reported during spaceflight, such as blurred vision, reduced contrast sensitivity, and overall retinal dysfunction [27,28]. However, because direct measurements of retinal Ca^2+^ dynamics under microgravity have not been reported, the relationship should currently be regarded as a mechanistic hypothesis rather than an established pathway.

#### 3.4.10. Lack of Direct Evidence for Ca^2+^ Disruption

While Ca^2+^ is crucial for retinal function, there are currently no studies that directly measure Ca^2+^ signaling disruption in retinal cells under microgravity conditions. Although Ca^2+^ imbalance has been investigated in other organ systems during spaceflight—such as the musculoskeletal and immune systems—there have been no in vivo or in vitro studies examining the effects of microgravity on Ca^2+^ concentrations or signaling pathways in retinal photoreceptors or neurons. This creates a clear gap in the literature. The impact of microgravity on Ca^2+^ regulation within the retina remains untested, and further studies are needed to determine whether Ca^2+^ signaling is directly affected by spaceflight exposure.

#### 3.4.11. Logical Link Between Ca^2+^ and Vision Dysfunction

Despite the lack of direct evidence, Ca^2+^ imbalance likely contributes to the visual changes observed in space. Ca^2+^ is essential for photoreceptor survival, synaptic signaling, and retinal homeostasis—as discussed in Section 1. In Section 2, we showed that microgravity causes structural and molecular damage to the retina. For example, Özelbaykal et al. (2022) reported retinal changes such as optic disc edema, thickening of the retinal nerve fiber layer, and signs of retinal stress in astronauts [29]. In Section 3, we demonstrated that Ca^2+^-binding proteins such as Calbindin, Calretinin, and Parvalbumin are downregulated under stress, weakening the retina’s ability to regulate intracellular Ca^2+^ concentrations [27].

While these specific changes have not yet been observed under microgravity conditions, they provide a logical explanation linking stress-induced retinal damage to potential Ca^2+^ imbalance. Collectively, current evidence demonstrates that microgravity produces structural, molecular, and functional alterations within the retina. However, no study has directly quantified retinal Ca^2+^ signaling under true or simulated microgravity conditions. Therefore, the hypothesis that microgravity-induced visual dysfunction is mediated, in part, by Ca^2+^ dysregulation remains inferential. Given the central role of Ca^2+^ in phototransduction, neuronal signaling, and retinal homeostasis, future studies should directly evaluate retinal Ca^2+^ dynamics during spaceflight and in validated microgravity analogs.

### 3.5. Touch

#### 3.5.1. Disruption of Touch Perception Through Ca^2+^ Signaling in Microgravity

Mechanosensitive ion channels, which are responsible for initiating the sensation of touch, such as Piezo and TRP channels, convert mechanical deformation at the surface of our skin into electrical and chemical signaling through the use of Ca^2+^ ion influx [30,31]. The TRP channels involved, such as TRPV4 and TRPA1, contribute to the detection of mechanical forces and allow for the adaptation of the touch response during sustained stimulation [32]. These mechanosensitive ion channels are embedded in the membranes of sensory neurons and peripheral tissues, where they regulate a subset of actions, which includes responding to stimuli such as pressure and stretch by allowing Ca^2+^ ions to enter [31]. This Ca^2+^ influx triggers a cascade of intracellular events that enable tactile perception and mechanotransduction [32]. However, because these pathways depend on Ca^2+^-mediated mechanotransduction, they may be vulnerable to microgravity-induced alterations in mechanical loading, although direct evidence remains limited.

#### 3.5.2. Ca^2+^ Signaling Breakdown and Impaired Tactile Precision in Microgravity

In microgravity, normal mechanical loading of the body is reduced. This reduction in mechanical loading may decrease the activation of mechanosensitive ion channels involved in tactile perception [30,31]. Because mechanosensitive ion channels contribute to Ca^2+^ influx during mechanotransduction, reduced channel activation could potentially alter intracellular Ca^2+^ signaling [30,31]. If Ca^2+^ influx is altered, intracellular signaling pathways involved in tactile transduction may also be affected, potentially contributing to reduced tactile sensitivity and precision under microgravity conditions [30,31]. Impairment of Ca^2+^ ion influx could also affect downstream processes, including synaptic transmission and neurotransmitter release, both of which are important for accurate sensory processing [30]. Specifically, Ca^2+^ forms a Ca^2+^- CaM complex with calmodulin (CaM) which can result in the activation of downstream signaling molecules such as CaMKII that are involved in touch signal adaptation and neuronal feedback regulation [33,34]. CaMKII is known for its role in activity-dependent synaptic plasticity, which aids in encoding sensory adaptation; it is particularly important for regulating feedback mechanisms during prolonged stimulation [34]. Therefore, if microgravity alters Ca^2+^ entry through mechanosensitive ion channels, downstream Ca^2+^-dependent signaling cascades may also be affected. This proposed mechanism may contribute to the tactile and sensorimotor deficits reported in microgravity analog studies, although direct experimental evidence linking altered Ca^2+^ signaling to these functional outcomes remains limited [35]. Therefore, current evidence should be interpreted as mechanistic and hypothesis-generating rather than demonstrating a direct causal relationship between microgravity and tactile Ca^2+^ dysregulation.

#### 3.5.3. Evidence from Dry Immersion and Head-Down Bed Rest Analog Studies

Dry immersion experiments described by Saveko et al. [35] replicated weightlessness by suspending participants in thermoneutral water using a waterproof membrane to manipulate Archimedes’ principle [35]. This experimental design reduced normal mechanical loading and support, reproducing several physiological features associated with microgravity exposure. The results demonstrated reduced tactile precision and impaired sensorimotor coordination under unloading conditions [35]. These findings suggest that unloading may impair tactile sensory processing; however, the underlying molecular mechanisms, including possible alterations in Ca^2+^-dependent mechanotransduction, were not directly examined [35]. Additionally, Saveko et al. [35] also did a head-down bed rest experiment, which simulates zero gravity by making the participant undergo a prolonged 6° tilt.

The results showcased impaired sensorimotor control and proprioceptive feedback [35]. These outcomes support the view that unloading conditions can impair tactile and sensorimotor function; however, direct evidence linking these effects to altered Ca^2+^ signaling remains limited. While these analog models provide controlled environments for testing microgravity’s effects on touch, direct data from spaceflight is limited. Therefore, the current understanding of tactile sensory disruption is primarily drawn from these simulations.

Direct evidence on tactile Ca^2+^ signaling in microgravity is limited, but analog models and parallels with other Ca^2+^-dependent sensory pathways indicate that mechanotransduction may be vulnerable in space. This theme continues in the auditory and vestibular systems, where hair cell transduction again depends on Ca and may be altered under conditions of weightlessness.

### 3.6. Hearing

#### 3.6.1. Role of Ca^2+^ in Hearing

The peripheral vestibular apparatus includes the cochlea, a snail-shaped structure located within the inner ear. Within the cochlea, the Organ of Corti sits on the basilar membrane and contains one row of sensory inner hair cells (IHCs) and three rows of sensory outer hair cells (OHCs). Together, these form the neurosensory epithelium necessary for auditory transduction [36].

Hair cells are arranged along the cochlea from base to apex in a tonotopic pattern. Hair cells near the base are shorter and respond to high frequencies, whereas those near the apex are longer and respond to low frequencies [36]. The epithelium is bathed in endolymph from the scala media, a unique extracellular fluid with a high concentration of K^+^ and a low concentration of Ca^2+^. This ionic composition establishes the electrochemical gradient necessary for mechano-electrical transduction. As a result, the ionic environment sets the stage for the process of mechanotransduction [37,38].

The process of encoding sound is known as mechanotransduction. It is achieved when sensory hair cells within the cochlea convert mechanical motion into electrical signals. These highly specialized mechanosensitive sensory hair cells are known as hair bundles, which consist of rows of stereocilia [37,38]. When sound waves enter the ear, they cause the fluid within the cochlea to move, bending the stereocilia and leading to the depolarization of hair cells [38]. This mechanical force opens mechano-electrical transduction (MET) channels on the stereocilia, allowing Ca^2+^ and K^+^ influx into the cell. Ca^2+^ then causes a rapid “reset” using Ca^2+^-dependent feedback mechanisms, which causes the MET channels to partially close within milliseconds. This allows fast adaptation, keeping the cells sensitive and preventing saturation [37,38].

#### 3.6.2. CaV1.3-Driven Neurotransmission at the Ribbon Synapse

The opening of MET channels within the stereocilia allows for Ca^2+^ and K^+^ influx, producing a receptor potential that depolarizes the hair cell. This depolarization spreads to the basolateral membrane where it will activate voltage-gated CaV1.3 channels in the ribbon synapse. Ca^2+^ entering through these CaV1.3 channels triggers synaptic vesicle fusion with the presynaptic membrane. This results in the release of glutamate, which activates auditory nerve fibers that transmit signals to the brain for processing [37,38]. Unlike many other voltage-gated Ca^2+^ channels, CaV1.3 exhibits sustained activation during prolonged hair cell depolarization, allowing continuous neurotransmission during ongoing stereocilia stimulation. Research from the University Medical Centre Göttingen and the Max Planck Institute for Multidisciplinary Sciences demonstrated that Ca^2+^-binding proteins CaBP1 and CaBP2 bind to Ca^2+^ and stabilize CaV1.3 channels in their open state during hair bundle stimulation [38]. Deletion of both the CaBP1- and CaBP2-encoding genes significantly impaired channel function. Without these proteins, the channels could not stay fully open. This reduced Ca^2+^ influx, altered glutamate release, and weakened activation of auditory nerve fibers. In genetically modified mice, these changes resulted in severe hearing loss [38]. These findings support the importance of Ca^2+^ signaling in maintaining CaV1.3 channel activity and enabling the transmission of neural signals from the cochlea to the brain. While the cochlea is specialized for detecting and transmitting sound, Ca^2+^ is equally critical in the otolith organs, which sense linear acceleration and head position within the vestibular system.

#### 3.6.3. Role of Ca^2+^ in the Otolith Organs

One of the critical roles Ca^2+^ has in the auditory and vestibular system is found in the otolith organs, which consist of the saccule and utricle. These gravity receptors are responsible for sensing the combined inertial force from linear head movement and head tilt relative to gravity. Both organs are structurally similar, consisting of an epithelium—the macula—which contains hair cells and supporting cells embedded in a vasoelectric gel layer called the otolithic membrane [39,40].

On the other end of the otolithic membrane lie crystals called otoconia, which are composed of Ca carbonate (CaCO_3_). Because of their mass, the otoconia are heavier than other surrounding structures, including the endolymph. Due to this heaviness, whenever the head tilts, the otoconia move relative to gravity, pulling on the otolithic membrane. This shear motion displaces the hair cells embedded within, bending them and causing a receptor potential. This can also occur when the head experiences linear acceleration.

The functional difference between both the saccule and the utricle can be understood through their anatomical orientations: the utricle is approximately in the horizontal plane when the head is upright, making it primarily sensitive to horizontal linear acceleration. In contrast, the saccule is oriented more vertically, making it more responsive to vertical acceleration and head tilt relative to gravity. When you lie fully flat, the acceleration causes displacement of the otolith membrane which bends the stereocilia altering hair cell signaling. Similarly, when you sit upright, the same forward push is picked up by the horizontally sensing utricle. Both organs respond to straight-line movement depending on head position, and by working together, they ensure consistent detection of linear head movement [39,40].

#### 3.6.4. Ca^2+^-Dependent Neurotransmission

Similar to the cochlea, Ca^2+^-dependent glutamate release drives nerve firing in the otolith organs. When the head experiences linear acceleration, the stereocilia of hair cells embedded within the otolithic membrane deflect relative to gravity. Head movement that causes the stereocilia to move away from the tallest hair cell, called the kinocilium, results in hyperpolarization of the hair cells. In contrast, movement of the stereocilia toward the kinocilium causes the hair cells to depolarize, opening Ca^2+^-dependent MET channels and allowing Ca^2+^ ions to flow into the cell [41,42].

This Ca^2+^ influx triggers glutamate release, which stimulates the vestibular afferent neurons. The resulting signals travel along the vestibular nerve to the central nervous system, where they are processed to determine motion and head position [41,42]. Given their reliance on Ca^2+^-dependent mechanisms, the otolith organs may be particularly vulnerable to alterations in Ca^2+^ homeostasis during microgravity exposure.

#### 3.6.5. Microgravity Effects on Hearing and Vestibular Function

Under normal conditions, otoconia exhibit a hexagonal symmetry [39]. Their high density increases the sensitivity of the otolith organs by pulling the otolithic membrane relative to gravity [39,43]. Experimental evidence suggests that microgravity-associated alterations in Ca^2+^ homeostasis may influence otoconia structure, potentially contributing to imbalance and spatial disorientation [39]. Hindlimb suspension models showed an increase in otoconia structure, whereas in-flight specimens demonstrated a decrease compared to Earth-based controls [39]. Spaceflight studies suggest that some organisms may compensate for the loss of gravity by upregulating CaCO_3_ deposition, which may increase otolith sensitivity to adapt to the weightless environment [39,40,44].

Although animal findings cannot be directly extrapolated to humans, they can provide a useful mechanistic insight into how altered gravity may affect otoconia structure and vestibular function, helping guide hypotheses about similar adaptations in astronauts. Studies on frogs and rats indicated that short-term microgravity exposure (seven days) increased otoconia mass [45]. Similarly, several aquatic and amphibious species raised in space—including the freshwater pond snail (Biomphalaria glabrata), marine mollusk (Aplysia californica), clawed frog (Xenopus), newt (Cynops pyrrhogaster), and swordtail fish (Xiphophorus helleri)—showed an increase in otoconia mass relative to controls raised on Earth [39].

This adaptive response may help regulate otolith function and could enhance sensitivity to allow normal vestibular performance in a weightless environment. Because the endolymphatic fluid surrounding the otolith organs has very low Ca^2+^ (~0.023 mM) and carbonate ion concentrations, adaptive mechanisms were likely necessary. These processes are thought to involve glycoproteins OC90 and otolin-1, which bind together to form a protein matrix that recruits and binds Ca^2+^ ions, facilitating CaCO_3_ crystallization on otoconia [44].

While this adaptation may be beneficial in space, prolonged microgravity may contribute to extended recovery times upon return to Earth. Post-flight changes may impair spatial orientation, balance, and perception of linear acceleration [45,46]. Understanding these otoconia adaptations is therefore critical for developing countermeasures that preserve vestibular function during and after long-term space missions. Alterations in otoconia morphology under loading conditions have been visualized and quantified using advanced imaging and elemental analysis techniques, as described in the following section.

#### 3.6.6. Scanning Electron Microscopy and X-Ray Microanalysis of Otoconia in Suspended Rats

**Figure 4:**
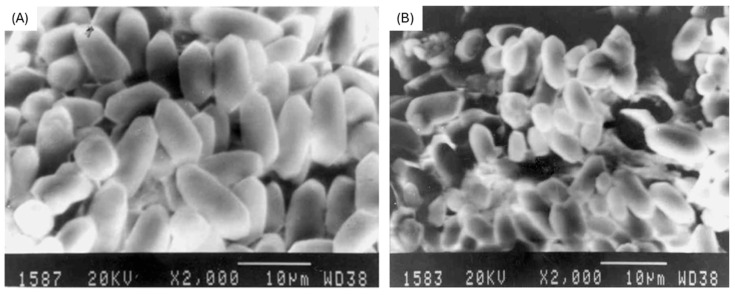
(**A**) SEM image of rat utricular/saccular otoconia (control) and (**B**) SEM image of rat utricular/saccular otoconia after 160-day tail-suspension model. Images reprinted from Ref. [47].

Using both scanning electron micrography (SEM), as well as X-Ray microanalysis, one study compared the utricle and saccule of both control group (housed individually) and an experimental group (rats suspended by the tail for 160 days) [47]. SEM images of the control group (Figure 4A) revealed the otoconia of the utricle and saccule were symmetrical, regularly shaped, tightly packed, and surfaces are shown to be smooth. In contrast, suspended rats (Figure 4B) exhibited irregularly shaped otoconia; some were out of order, some crystals resembled small balls which had a rough surface [47]. These findings suggest that in prolonged unloading conditions this can alter mineral organization as well as otoconia morphology. However, although hindlimb suspension models reproduce aspects of unloading and fluid redistribution, they do not fully simulate the spaceflight environment or conditions of true microgravity. Elemental analysis (X-ray Microanalysis), in both control and suspended rats, identified peaks of Ca^2+^ as well as other ions. Ca^2+^ content was calculated using the number of photons and the photon beam intensity [47]. This is summarized in Table 1 showcasing both groups (suspended for 160 days and control) Ca^2+^ content of saccule, utricle as well as the ossicles.

#### 3.6.7. Ca^2+^-Dependent Neurotransmission in Otolith Organs

Although little is known about the specific effects of microgravity on hair cell function within the otolith organs, a potential mechanism can be proposed. In the absence of the usual gravitational vector acting on the otoconia, the mechanical loading of the otolithic membrane may be reduced. As a result, the magnitude of stereociliary deflection may be altered, potentially reducing Ca^2+^ influx and modifying glutamate release. Reduced glutamate release may alter vestibular afferent firing, potentially disrupting signals to the central nervous system (CNS) for processing.

These alterations may contribute to a conflict between actual and anticipated sensory signals. As a result, spatial orientation and balance in astronauts may be impaired [46]. Such alterations could also explain the occurrence of space motion sickness. Impairment on this scale can make even simple tasks challenging during spaceflight.

#### 3.6.8. Changes in Cochlear Function Under Microgravity

Compared with vestibular adaptations, direct evidence describing cochlear Ca^2+^ dysregulation under microgravity remains limited. Under normal conditions, mechanical forces within the fluid-filled inner ear deflect vestibular hair bundles, thereby activating vestibular receptor hair cells and supporting vestibular signaling [48]. This allows for precise hair cell stimulation and Ca^2+^-dependent neurotransmission. However, during spaceflight, altered gravitational loading and fluid redistribution may contribute to inner ear dysfunction and loss, or a sensation of fullness in the ears of astronauts, although the underlying cochlear mechanisms remain unclear [46,49]. These changes could also contribute to impaired balance and spatial orientation during microgravity exposure [46].

Based on available evidence, we hypothesize that while microgravity’s effects on the morphology of otoconia are well documented, the same cannot be said about how weightlessness alters Ca^2+^-dependent neurotransmission in the cochlea. Most current reports describe subjective symptoms (ear fullness and temporary hearing loss) rather than cellular-level mechanisms. A proposed mechanism is that the disruption of fluid movement may cause uncoordinated stereocilia deflection. This could potentially alter Ca^2+^ influx through MET channels, potentially impairing Ca^2+^-dependent fast adaptation. Prolonged MET channel opening could cause hair cells to become less sensitive. This could explain the transient hearing changes, highlighting the need for in-flight electrophysiological studies to verify the mechanism and develop prevention strategies. Taken together, current evidence suggests that microgravity may influence Ca^2+^ homeostasis across five senses.

**Figure 5:**
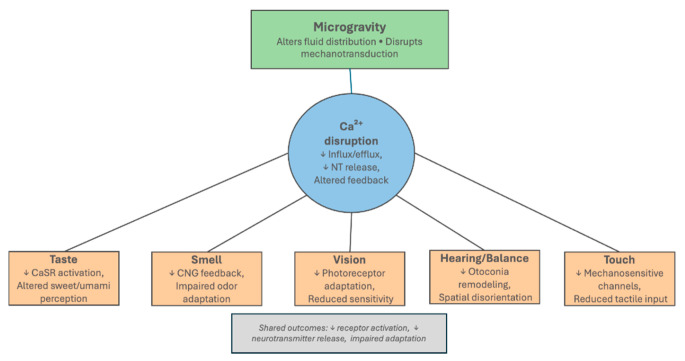
Schematic summary of microgravity-induced Ca^2+^ signaling disruption across the human senses.

A summary of microgravity-induced Ca^2+^ signaling disruption and its downstream effects on the five sensory systems is shown in Figure 5. Microgravity disrupts Ca^2+^ signaling, impairing influx/efflux regulation, neurotransmitter release, and feedback mechanisms. These changes cascade across sensory systems, leading to deficits in taste, smell, vision, hearing/balance, and touch [19,20,21,26].

## 4. Discussion

### 4.1. Summary of Key Findings

This review assembles cellular, molecular, and physiological evidence to examine how microgravity disrupts Ca^2+^ signaling across the five primary sensory systems in the body. Across all five senses, evidence suggests that microgravity may disrupt Ca^2+^-dependent mechanisms. These shared patterns highlight the central role of Ca^2+^ signaling in sensory function and suggest that it may represent a common pathway affected by microgravity. Collectively, these findings suggest that sensory problems observed during microgravity may be influenced by a common underlying disruption, rather than separate mechanisms within each individual sense.

### 4.2. Integration with Existing Literature

Past research has demonstrated that reduced gravity conditions may be associated with disturbed Ca^2+^ homeostasis in multiple tissues [19,20,26,27]. However, most previous studies addressed individual senses or solitary pathways [16,22,29].

In taste, the proposed impairment of CaSR function is in line with studies by Gruener et al. [11], which described spaceflight-related myocyte membrane disruptions. These membrane disruptions may indicate a broader vulnerability of Ca^2+^-sensitive receptor systems involved in taste perception [11]. Similar ionic disturbances have been reported for sublingual epithelial cells from Rhesus monkeys [12]; therefore supporting the possibility of disrupted Ca^2+^ homeostasis affecting taste. While some external factors like decreased airflow and changed food plating impact taste [15,22], they may act alongside astronauts’ molecular level changes. However, direct evidence on Ca^2+^’s role in gustation during spaceflight is scarce, and much of the current understanding relies on analog models and mechanistic inference. This gap highlights a priority area for future research, given the importance of taste perception for astronaut nutrition and well-being.

Olfactory results are consistent with Reisert and Reingruber’s [17] studies on the requirement of Ca^2+^-activated chloride channels for signal integrity, as well as Takeuchi and Kurahashi’s [16] findings regarding Ca^2+^’s dual involvement in amplifying smell responses and enabling adaptation. The documented downregulation of calmodulin and CAMKII in microgravity [21] may be consistent with reduced adaptive capacity in olfactory processing, while the structural alterations of the olfactory bulb [23] may further contribute to delayed recovery or persistent olfactory alterations [23].

In the visual sense, findings are consistent with Mao et al.’s [26] observations of microgravity-induced oxidative stress and apoptosis in retinal tissue [26]. Expression of CLIP2 and CBP [27] has also been shown to be altered in the retina in the diabetic and ischemic retinopathies [27,28] which raises the possibility that spaceflight might speed up the age-related changes in the retina of normal eyes.

In the tactile system, decreased mechanosensitive channel activity in microgravity analogs [35] is consistent with the strong Ca^2+^ dependence of tactile mechanotransduction, suggesting that reduced channel activity may be associated with weaker sensory encoding [30,31]. These changes may contribute to the reduced tactile acuity noted in astronauts. Still, the evidence base for tactile Ca^2+^ signaling in microgravity is limited, with most findings drawn from ground-based analogs rather than real spaceflight. Because fine touch is essential for astronaut dexterity and motor coordination, this remains an important research gap.

For the auditory and vestibular systems, evidence such as changed otoconia structure and Ca^2+^ depletion [49], suggest impaired inertial sensing. Despite the lack of exploration of cochlear Ca^2+^ signaling, parallels with vestibular disruption and the Ca^2+^ dependence of hair cell transduction [2,34,44] suggest that auditory pathways may also be sensitive to microgravity-induced alterations in Ca^2+^ signaling.

An important consideration when interpreting these findings is that many of the investigations relied on microgravity analog models rather than actual spaceflight exposure. Models such as head-down bed rest, dry immersion, reclined posture paradigms, and virtual reality environments reproduce selected physiological or perceptual aspects of spaceflight, including fluid changes, unloading, or sensorimotor conflict. However, they do not fully reproduce the multifactorial conditions seen during true space missions, including prolonged exposure to microgravity, radiation, confinement, and disturbed circadian cycles. As a result, conclusions drawn from these analogs should be regarded with caution and might not apply directly to humans in space. Future investigations combining analog models with in-flight studies will be essential to validate the role of Ca^2+^ signaling in sensory adaptation during spaceflight.

In addition to limitations related to microgravity analog models, sensory changes during spaceflight should not be linked to Ca^2+^ signaling alone. Spaceflight creates a multifactorial physiological and environmental setting in which several non-Ca^2+^ contributors may affect sensory perception, including cephalad fluid shifts, nasal/sinus congestion, altered airflow, elevated CO_2_ levels, stress, sleep disruption, diet, food monotony, food packaging, and the confined spacecraft environment. Hodkinson et al. describe spaceflight as a physiologically challenging environment and report early fluid redistribution from the lower to upper body, facial edema or “puffy face”, a 10–15% reduction in plasma volume, nasal/sinus congestion, increased CO_2_ as a space-affiliated differential factor, and significant sleep disruption caused by altered light/dark cycles, illumination and crew workload [1]. Similarly, Prabodha et al. emphasize that food and flavor perception in space are shaped by interacting physiological, psychological, environmental and sensory factors, including an altered appetite, food preferences, food monotony, rehydration methods, water quality, and multimodal interactions between taste, smell, texture, vision and the surrounding environment [2]. Therefore, Ca^2+^ signaling should be interpreted as one possible cellular and molecular mechanism that may contribute to sensory disruption, rather than the sole causal pathway responsible for sensory alterations during spaceflight. These non-Ca^2+^ factors may act independently or interact with Ca^2+^-dependent pathways, so the causal framework should remain broad and cautious.

### 4.3. Clinical Implications

From a patient-care perspective, these findings may help inform future therapeutic strategies aimed at addressing sensory dysfunction associated with altered Ca^2+^ signaling.

These findings could be translated into clinically actionable strategies to create therapeutic options for patients who may experience these symptoms. Mechanism-oriented methods to pinpoint particular changes in Ca^2+^-signaling pathways linked to sensory impairment may be helpful in future research. When an altered protein or pathway is found in one of the five senses, one potential approach for investigation is targeted modulation, which is the use of selective receptor ligands or other regulators for Ca^2+^ signaling, which aims to restore Ca^2+^ homeostasis whether on Earth or on spaceflight. Depending on the sensory system affected, future management approaches could potentially be tailored to improve efficiency, outcomes, and reduce complications.

If future studies establish clinically relevant alterations in Ca^2+^ signaling, assessment strategies such as audiometry, olfactory/gustatory tests, as well as somatosensory evaluations may help characterize the affected sensory systems. Should specific Ca^2+^-signaling pathways be identified as contributors to sensory dysfunction, treatment plans (medications, procedures, and rehabilitation) may be tailored according to the affected sensory modality and underlying molecular mechanisms. Overall, these findings may contribute to the development of future countermeasures for sensory dysfunctions in both terrestrial and spaceflight settings.

However, it is important to note that many of these clinical strategies remain theoretical, as direct evidence linking Ca^2+^-targeted interventions to sensory outcomes during spaceflight is limited. Careful validation will be required before clinical translation.

### 4.4. Direct Astronaut Relevance

The sensory disruptions described above may have important implications for astronaut safety, performance, and mission outcomes. Each sensory system is connected to critical operational tasks, and even the most subtle Ca^2+^-related impairments could worsen safety concerns and mission performance.

## 5. Future Directions

Moving forward, priority should be given to studies that directly measure Ca^2+^ dynamics in human sensory tissues during spaceflight, as these represent one of the most significant current knowledge gap. Where feasible, future studies should incorporate real spaceflight experiments to complement findings from ground-based analogs such as head-down bed rest and dry immersion [35]. However, feasibility is constrained due to limited spaceflight opportunities, high costs, restricted astronaut time, and operational requirements that affect sampling and experimental complexity. Ethical and safety considerations require that such studies be noninvasive and low-risk and must not compromise the crew or mission performance. A possible solution would be to use a staged pipeline that utilizes human-relevant in vitro systems and complementary in vivo models to refine hypotheses and lower risk before targeted in-flight experimentation [19,20,21]. Non-invasive neuroimaging approaches like EEG should also be utilized to study how altered Ca^2+^ dynamics influence real-time sensory processing in microgravity [19,27].

Future research should also delve into individual variability in Ca^2+^ signaling due to genetic or epigenetic differences [9,10]. Equally important will be cross-sensory studies that examine how disruptions in one modality, such as vision or vestibular function, interact with other senses [22,30,49]. Future research may also explore potential countermeasures aimed at supporting Ca^2+^ signaling pathways, including pharmacological modulation of mechanosensitive and Ca^2+^-dependent signaling systems such as TRP channels [32]. Other promising strategies include artificial gravity protocols [26,35] and dietary interventions aimed at supporting Ca^2+^ homeostasis [12,39,44]. These developed countermeasures should first be tested using analogs and then validated during spaceflight [35,45]. Ultimately protecting astronauts’ sensory processing through such countermeasures is vital not just for technical execution but also for psychological resilience, wellbeing, and mission success during long duration spaceflight [13,22,31].

In summary, further investigation of Ca^2+^ signaling as a potential unifying mechanism of sensory adaptation in microgravity may advance our understanding of space medicine and help guide the development of evidence-based countermeasures for long-duration missions.

## 6. Conclusions

In conclusion, this review highlights Ca^2+^ signaling as a potential unifying mechanism underlying sensory vulnerability in microgravity. Evidence across multiple sensory modalities suggests that microgravity may affect Ca^2+^-dependent processes involved in sensory transduction, neuronal signaling, and adaptive responses. However, the strength of evidence varies considerably between sensory systems, and direct demonstrations of microgravity-induced Ca^2+^ dysregulation remain limited in several modalities. While findings from spaceflight studies, analog environments, and related physiological models support a role for altered Ca^2+^ signaling in sensory adaptation, many of the proposed mechanisms remain hypothetical and require further experimental validation. As such, Ca^2+^ dysregulation should be viewed as a promising framework for understanding sensory changes in microgravity rather than as an established mechanism across all sensory systems. Future research should focus on directly measuring Ca^2+^ dynamics within sensory tissues during spaceflight and determining whether alterations in Ca^2+^ signaling are causally linked to sensory dysfunction. Addressing these knowledge gaps will strengthen our understanding of sensory adaptation in space and may help guide the development of targeted countermeasures aimed at preserving astronaut performance, health, and mission success during long-duration exploration missions.

## Figures and Tables

**Table 1:** Ca^2+^ content (mean ± SD; counts per second, CPS) in utricle, saccule, and stapes from control vs tail-suspended rats. Table reprinted from Ref. [47].

Source	Control Group	Suspended Group	*p*
Utricle	4350.50 ± 894.40	1855.50 ± 432.20	≤0.01
Saccule	1443.17 ± 280.23	946.44 ± 289.07	≤0.01
Ossicle (stapes)	10,744.00 ± 2050.11	7888.17 ± 887.39	≤0.01

## Data Availability

No new data is generated in this review article.
